# Autologous Mesenchymal Stroma Cells Are Superior to Allogeneic Ones in Bone Defect Regeneration

**DOI:** 10.3390/ijms19092526

**Published:** 2018-08-25

**Authors:** Anna E. Rapp, Ronny Bindl, Annika Erbacher, Anne Kruchen, Markus Rojewski, Hubert Schrezenmeier, Ingo Müller, Anita Ignatius

**Affiliations:** 1 Institute of Orthopaedic Research and Biomechanics, Trauma Research Center Ulm, University of Ulm, 89081 Ulm, Germany; anna.rapp@drfz.de (A.E.R.); ronny_bindl@web.de (R.B.); 2 Department of General Paediatrics, Haematology, and Oncology, University Children’s Hospital Tübingen, 72076 Tübingen, Germany; Annika.erbacher@uni-tuebingen.de; 3 Division for Pediatric Stem Cell Stem Cell Transplantation and Immunology, University Medical Centre Hamburg-Eppendorf, 20246 Hamburg, Germany; a.kruchen@uke.de (A.K.); i.mueller@uke.de (I.M.); 4 Institute of Clinical Transfusion Medicine and Immunogenetics, German Red Cross Blood Transfusion Service, Baden Wuerttemberg-Hessen and University Hospital Ulm, 89081 Ulm, Germany; markus.rojewski@uni-ulm.de (M.R.); h.schrezenmeier@blutspende.de (H.S.); 5 Institute of Transfusion Medicine, University of Ulm, 89081 Ulm, Germany

**Keywords:** bone regeneration, large bone defect, humanized mouse, allogeneic, stem cells, MSC

## Abstract

The application of autologous mesenchymal stem cells (MSC) for the treatment of bone defects requires two invasive procedures and several weeks of ex vivo cell expansion. To overcome these limitations, the administration of allogeneic MSC may be attractive, because they are anticipated to be immunoprivileged. Because preclinical studies using various animal models are conflicting with respect to the efficacy of allogeneic MSC, we investigated whether autologous and allogeneic human MSC (hMSC) are equally effective in regenerating bone in a humanized mouse model resembling the human immune system. Applying autologous and allogeneic hMSC in critically sized femoral defects, we found that allogeneic hMSC elicited a mild immune response early after implantation, whereas early angiogenic processes were similar in both treatments. At later healing time points, the transplantation of allogeneic hMSC resulted in less bone formation than autologous hMSC, associated with a reduced expression of the osteogenic factor Runx2 and impaired angiogenesis. We found by species-specific staining for collagen-type-1α2 that MSCs of either source did not synthesize new bone matrix, indicating an indirect contribution of transplanted hMSC to bone regeneration. In conclusion, our data suggest that the application of autologous hMSC is superior to that of allogeneic cells for bone defect treatment.

## 1. Introduction

Autologous stem-cell-based regenerative therapies for bone defect regeneration have a long history of clinical application. Thirty years ago, whole bone marrow (BM) was being successfully applied to treat delayed bone healing [1,2]. Percutaneous injection of concentrated BM into tibial non-unions resulted in bony consolidation of the defect in 88% of patients [3]. Concentration of BM leads to higher numbers of mesenchymal stroma cells (MSC) per mL compared to unprocessed BM and, interestingly, the authors found a positive correlation between fibroblast colony-forming units, i.e., MSC, in the injected BM preparation and the volume of the mineralized callus. Recently, ex vivo expanded autologous BM-derived MSC were shown to be safe and effective for the treatment of long-bone non-unions in a multicentric European study [4]. Additionally, the efficacy of autologous stem cells to regenerate bone was confirmed in pre-clinical models [5,6].

The isolation and ex vivo expansion of MSC to achieve clinically relevant cell numbers are time-consuming. Therefore, the application of allogeneic MSC, that is, cells from another donor that could be used off the shelf, could be an interesting alternative, because these cells are anticipated to be immunoprivileged and even immune-regulatory [7]. Clinically, allogeneic BM and allogeneic BM-derived MSC were successfully transplanted into children suffering from *osteogenesis imperfecta* [8,9,10]. Following treatment, the children displayed accelerated growth velocity and improved osteogenesis. Of note, the cell donors were carefully selected to be human leucocyte antigen-identical or single-mismatched, and some children received myeloablative treatment [9]. Pre-clinical studies on the use of non-autologous MSC to regenerate bone defects showed heterogeneous results. Successful use of allogeneic MSC for bone regeneration was reported in rabbits, sheep, and dogs [11,12,13]. In a more recent study, equivalent bone formation was reported when combining synthetic scaffolds with autologous or allogeneic MSCs in an ovine model [14]. However, others reported inferior bone formation, accompanied by increased cellular reactions or increased Th1 cytokine levels and decreased osteogenic differentiation markers when allogeneic or even xenogeneic cells were applied [6,15,16,17]. Collectively, the literature on the efficacy of non-autologous MSC for bone regeneration is inconclusive, thus warranting further studies. Furthermore, from the literature, it appears as though the model organism used for the studies critically influences the success of allogeneic MSC treatment, and conclusions for human systems are difficult to draw. Therefore, we investigated whether allogeneic and autologous human MSC (hMSC) are equally effective in the consolidation of large bone defects in a mouse model with a humanized immune system [18] that has not previously been used for studies on bone regeneration. We chose this model for our investigation, because it might help narrow the gap between preclinical models and the human situation.

## 2. Results

### 2.1. The Bone-Healing Capacity of Humanized Mice Is Not Significantly Affected by the Humanization Procedure

**Figure 1:**
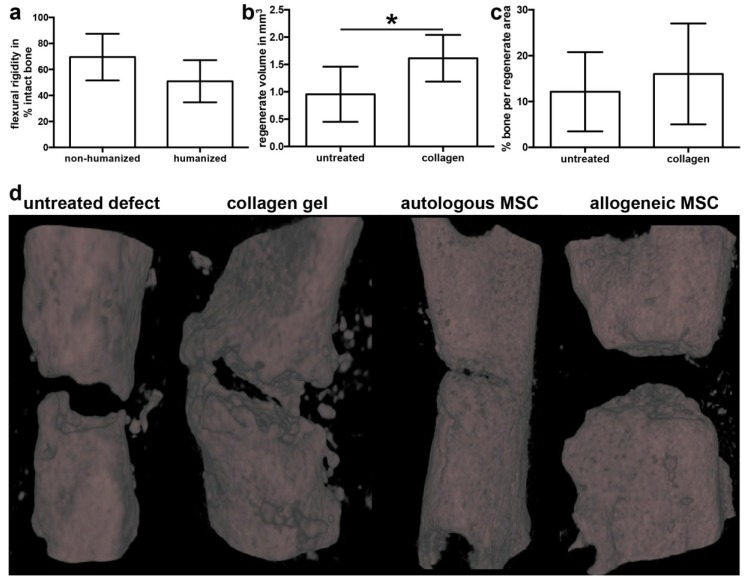
Validation of the defect model. (**a**) Stiffness (flexural rigidity) of healed osteotomies in non-humanized and humanized (*n* = 6) NOD/scid-IL2Rγ_c_^null^ mice relative to the intact femur. (**b**) Volume of the regenerate in mice with untreated defects and defects treated with cell-free collagen assessed by micro-computed tomography (µCT). (**c**) Analysis of the bone fraction in the regenerate in untreated and collagen-filled defects by µCT. (**d**) Representative three-dimensional reconstructions of untreated defects and defects filled with cell-free collagen, collagen with autologous human mesenchymal stem cells (hMSC), or collagen with allogeneic hMSC. All analyses were performed on day 35 after surgery. The data are presented as the mean ± SD, * *p* ≤ 0.05. non-humanized; *n* = 8; humanized *n* = 6.

To validate our model, we assessed the intrinsic bone-healing capacity of humanized mice to heal a non-critical bone injury to exclude effects of irradiation and transplantation of human hematopoietic cells. For this, we created transverse osteotomies that were stabilized using an external fixator in the femur of humanized NOD/scid-IL2rγ_c_^null^ (NSG) mice and compared the healing outcome with non-humanized NSG mice. We reported previously that immunodeficient NSG mice are able to heal bony injuries in an adequate time, although their healing was delayed as compared to immunocompetent Balb/c mice [19]. Here, we found a slight but nonsignificant decrease in the relative flexural rigidity of the humanized mice compared to non-humanized NSG (Figure 1a), indicating no effect of the humanization procedure on the intrinsic healing capacity.

Next, we created 1-mm defects that were left untreated or filled with a cell-free collagen type 1 gel. After 35 days, the tissue volume assessed by micro-computed tomography (µCT) was significantly increased in mice that received cell-free collagen gel compared to empty defects (Figure 1b). The relative bone fraction within the defect region was not significantly different between untreated and collagen-filled defects (Figure 1c). Representative three-dimensional reconstructions of all treatment groups, including hMSC treatment, are depicted in Figure 1d. Histologically, we found typical atrophic non-unions with closed or almost closed cortical ends in mice with untreated defects after a period of 35 days. In defects filled with cell-free collagen gel, little bone formation was evident, but no bridging of the defect (Figure 1d). Therefore, our model is valid to investigate the regenerative capacity of transplanted MSC.

### 2.2. Implantation of Autologous hMSC Results in Significantly More Bone Formation Compared to Allogeneic hMSC

**Figure 2:**
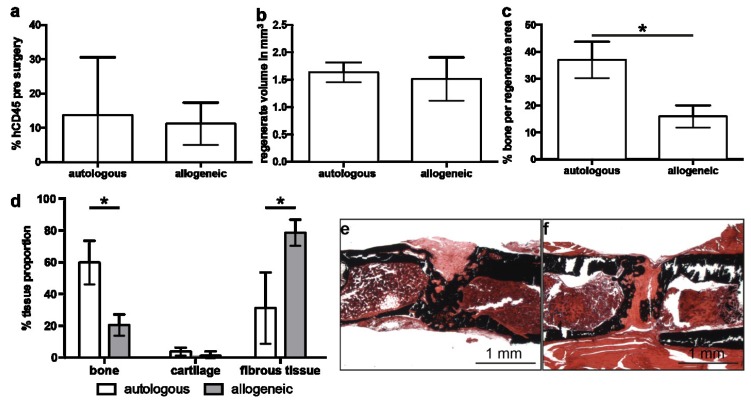
Analysis of defect regeneration in mice treated with autologous or allogeneic human mesenchymal stem cells (hMSC). (**a**) Percentage of human CD45-positive cells in the blood of humanized mice pre-surgery analyzed by flow cytometry. (**b**) Volume of the regenerate and (**c**) bone fraction in the regenerate assessed by micro-computed tomography on day 35 after surgery. (**d**) Analysis of the regenerate composition by histomorphometry on day 35. (**e**,**f**) Representative von Kossa-stained sections through the regenerate area of mice treated with autologous (**e**) and allogeneic (**f**) hMSC. The data are presented as the mean ± SD, * *p* ≤ 0.05. In (**a**) autologous *n* = 6, allogeneic *n* = 9. In (**b**–**d**) autologous *n* = 4, allogeneic *n* = 8.

Prior to surgery, we analyzed the number of human CD45^+^ lymphocytes in the blood to verify successful humanization. There was no significant difference between mice designated to autologous or allogeneic hMSC treatment (Figure 2a), such that the conditions at the time of surgery were the same. At 35 days after MSC implantation, µCT analysis revealed similar tissue volumes in the former defect region in both cell-treated groups (Figure 2b). However, we found significantly more bone in the regenerate of mice treated with autologous hMSC compared to treatment with allogeneic hMSC (Figure 2c). Histological analysis and histomorphometry confirmed these observations: in the autologous hMSC treatment group, the defect region was almost completely filled with newly formed bone bridging the cortices, whereas the defect in mice treated with allogeneic hMSC was filled with fibrous tissue and presumably remaining collagen gel (Figure 2d–f). The cartilage content did not differ significantly between the cell-treated groups.

### 2.3. Allogeneic hMSCs Elicit a Mild Cellular Immune Response

**Figure 3:**
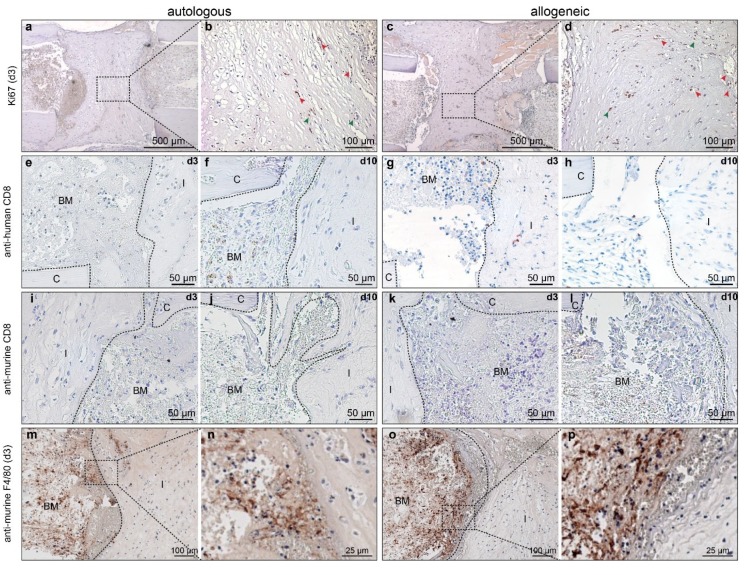
Immunohistochemical analysis of cell proliferation and inflammatory cells. (**a**–**d**) Staining of Ki67 on day 3 in mice with (**a**,**b**) autologous human mesenchymal stem cells (hMSC) treatment, and (**c**,**d**) with allogeneic hMSC treatment. (**e**–**h**) Staining for human CD8 on (**e**,**g**) day3 and (**f**,**h**) day 10 in mice with (**e**,**f**) autologous hMSC treatment and (**g**,**h**) allogeneic hMSC treatment. (**i**–**l**) Staining for murine CD8, the respective days are indicated in the upper right corner of the micrographs. (**m**–**p**) Staining of murine F4/80 in mice with (**m**,**n**) autologous hMSC treatment and (**o**,**p**) allogeneic hMSC treatment. BM: bone marrow; FT: fibrous tissue; C: cortical bone; I: implant. Dashed boxes indicate areas displayed in higher magnification. Red arrowheads in (**b**,**d**) indicate positive cells within vessel-like structures; green arrowheads indicate positive cells in the collagen gel matrix.

Staining for the proliferation marker Ki67 revealed positive cells within the gel matrix on day 3 after implantation (Figure 3a,c). The positive signal for Ki67 frequently co-localized with vessel-like structures (Figure 3b,d; red arrowheads), suggesting that these cells might be circulating immune cells, while only some cells embedded in the matrix appeared to be stained (Figure 3b,d, green arrowheads). To elucidate whether immunological reactions early during the regeneration process differed in after the implantation of allogeneic or autologous hMSC, respectively, we analyzed the response at day 3. Systemically, we found no differences in the serum levels of the pro-inflammatory mediators keratinocyte chemoattractant (CXCL-1), monocyte chemotactic protein (MCP)-1, interleukin (IL)-6, IL-1β, and tumor necrosis factor (TNF)-α between the treatment groups on day 3 after MSC implantation (Table 1). Similarly, the level of anti-inflammatory IL-4 was not different (Table 1). The mediators interferon (IFN)-γ, macrophage inflammatory protein (MIP)-1α, IL-10, and IL-13 were not detectable. To investigate local inflammatory processes, we stained sections for human and murine CD8^+^ T cells and for murine macrophages. As expected, autologous MSC did not elicit an immune response on days 3 or 10 after implantation (Figure 3e,f). However, in mice treated with allogeneic MSC, we found human CD8^+^ cells near the cell-seeded collagen implant at both time points, indicating a mild immune response towards the MSC (Figure 3g,h). Staining for murine CD8 was negative regardless of the time point or cell source (Figure 3i–l). Murine F4/80-positive cells were detected in both treatment groups to a similar extent (Figure 3m–p).

### 2.4. Early Angiogenesis Was Not Different between Mice Treated with Autologous or Allogeneic hMSC

**Figure 4:**
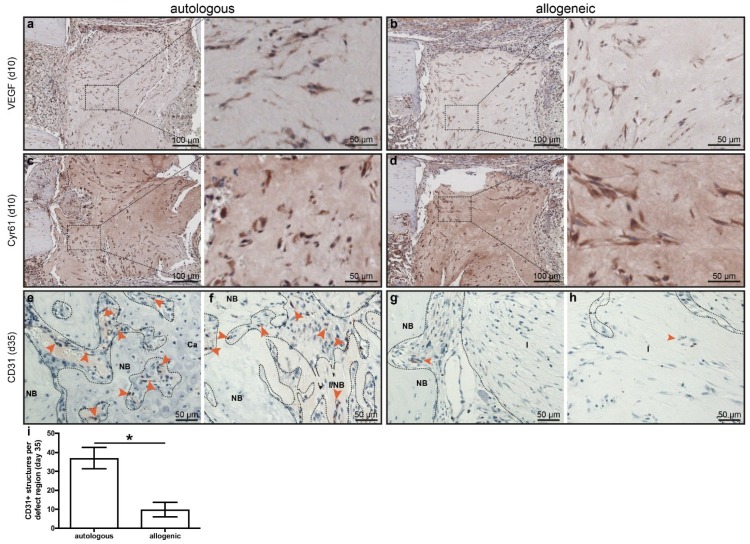
Expression of angiogenic markers. (**a**,**b**) Staining of vascular endothelial growth factor (VEGF) on day 10 in (**a**) mice with autologous human mesenchymal stem cells (hMSC) treatment and (**b**) mice with allogeneic hMSC treatment. (**c**,**d**) Staining for Cyr61 on day 10 in mice with (**c**) autologous hMSC treatment and (**d**) allogeneic hMSC treatment. (**e**–**h**) Staining of CD31 (PECAM) on day 35 in mice with (**e**,**f**) autologous hMSC treatment and (**g**,**h**) allogeneic hMSC treatment. (**e**) and (**g**) represent the edge of the implant while (**f**,**k**) represent the center of the implant. NB: new bone; Ca: cartilage, I: implant. Arrowheads indicate structures positive for CD31. (**i**) Quantification of CD31 staining. Dashed boxes indicate areas displayed in higher magnification. The data are presented as the mean ± SD, * *p* ≤ 0.05, autologous *n* = 2; allogeneic *n* = 6.

Next, we analyzed the presence of angiogenic factors and the formation of new vessels within the defect region, because an adequate supply of oxygen and nutrients is essential for bone formation. On day 10, there were no differences in the staining for the angiogenic factors vascular endothelial growth factor (VEGF) and Cyr61 in mice treated with autologous or allogeneic MSC (Figure 4a–d). Staining for murine CD31 revealed positively stained structures on day 10 in the surrounding of defect region in both mice treated with autologous or allogeneic MSC with no differences (data not shown). However, at a later stage of day 35, there were significantly more CD31^+^ structures evident in defects treated with autologous MSC compared to defects treated with allogeneic MSC (37.0 ± 4.0 vs. 9.8 ± 1.6 structures/mm^2^; Figure 4e–i). In mice treated with allogeneic MSC, some vessels could be found on the edges of the defect (Figure 4g), but scarcely in the center (Figure 4h), while there were numerous CD31^+^ structures throughout the whole defect region in mice treated with autologous hMSC (Figure 4e,f).

### 2.5. Human Cells Are Present Up to 35 Days in Defects Treated with hMSC, but These Do Not Synthesize Bone Matrix

**Figure 5:**
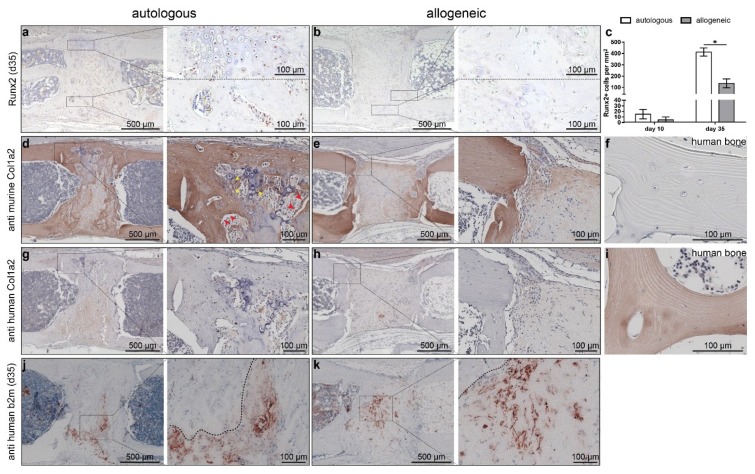
Analysis of osteogenesis by autologous and allogeneic human mesenchymal stem cells (hMSC) on day 35. (**a**–**c**) Staining of Runx2 on day 35 (**a**,**b**) and quantification (**c**) in mice with (**a**) autologous hMSC treatment and (**b**) allogeneic hMSC treatment. (**d**–**f**) Staining for murine collagen type 1α2 in (**d**) mice with autologous hMSC treatment, (**e**) mice with allogeneic hMSC treatment, and (**f**) human control tissue. (**g**–**i**) Staining for human Col1α2 in mice with (**g**) autologous hMSC treatment, (**h**) allogeneic hMSC treatment, and (**i**) human bone. (**j**,**k**) staining for human β2-microglobulin (b2m). Dashed boxes indicate areas displayed in higher magnification. The data are presented as the mean ± SD, * *p* ≤ 0.05, *n* = autologous *n* = 2; allogeneic *n* = 3. Red arrowheads in (**d**) indicate bone lining cells positive for murine Col1α2; yellow arrowheads indicate cells within hypertrophic cartilage positive for murine Col1α2.

To determine whether the implanted hMSC persist over the entire observation period, we stained sections for human β2-microglobulin (hb2m). A positive signal was observed until day 35 in both the allogeneic- and autologous-treated mice in what appeared to be residual collagen gel. Of note, cells embedded into the newly formed bone were negative for hb2m (Figure 5j,k). To clarify whether the implanted hMSC directly contribute to new bone formation, we stained adjacent sections for the species-specific collagen type I alpha 2 chain (Col1α2) using anti-murine and anti-human Col1α2 antibodies. Reflecting the findings from µCT (Figure 2), a larger area was positive for Col1α2 in mice that received autologous hMSC (Figure 5d,e). We found intensive staining for murine Col1α2 (Figure 5d,e), but no staining for human Col1α2 irrespective of MSC origin (Figure 5g,h). In mice treated with autologous MSC, it was clearly observed that cells lining the new bone were positive for murine Col1α2 (Figure 5d; red arrowheads) and that the collagen that was deposited within the hypertrophic cartilage was of murine origin (Figure 5d; yellow arrowheads). The same areas were not stained when the anti-human Col1α2-antibody was applied (Figure 5g,h). Human control tissue was negative for murine Col1α2 (Figure 5f). The dense fibrous tissue that we observed predominantly in mice with allogeneic hMSC treatment was negligibly stained for mCol1α2, indicating no remodeling of the implanted collagen gel in the absence of bone formation (Figure 5h). For further analysis of bone formation, we investigated osteogenic-marker expression by immunohistochemistry. While there was no difference in Runx2 staining on day 10, significantly more Runx2-positive cells were evident in defects treated with autologous hMSC after 35 days, indicating more advanced osteogenesis (Figure 5a–c).

Overall, our results suggest a delayed and diminished osteogenic differentiation and osteogenesis when allogeneic MSC were applied, as well as an overall reduced regenerative capacity of allogeneic hMSC.

## 3. Discussion

Autologous MSCs are already applied for bone regeneration in clinics. However, the autologous approach requires an additional invasive procedure to obtain bone marrow aspirate for cell isolation, and treatment is available only with some delay due to the two- to three-week ex vivo expansion period in order to achieve clinically relevant cell numbers. Therefore, the application of allogeneic hMSC as an “off-the-shelf” product appears to be an attractive alternative. However, our results in a mouse model with a humanized immune system indicated an inferior efficacy of allogeneic hMSC to regenerate bone compared to autologous cells. We found that implantation of autologous hMSC led to almost complete bridging of the bone defect, whereas there was no considerable bone formation evident in mice that were treated with allogeneic hMSC. While there were no signs of systemic inflammation or defective early neo-angiogenesis, we found a local increase in CD8^+^ T cell numbers in allogeneic-treated mice and reduced Runx2 expression.

Confirming superior bone formation by autologous MSC, Coathup et al. detected greater amounts of bone surrounding tumor prosthesis when these were spray-coated with autologous MSC compared to coating with allogeneic MSC, although the underling mechanism was not investigated [6]. Chatterjea and colleagues reported superior bone formation of osteogenically primed syngeneic MSC—which can be considered as autologous—compared to allogeneic MSC in a rat model [20]. Similar to our results, the authors demonstrated the recruitment of T cells to the implanted allogeneic cells. Administration of an immunosuppressive agent over a prolonged time resulted in bone formation by allogeneic MSC from one donor, whereas cells of another donor would still not form any bone, indicating complex, yet not fully understood mechanisms [20]. The importance of the immunological response for successful bone regeneration has also been recognized by others. Dighe et al. found impaired bone formation in an ectopic model when allogeneic Balb/c-derived MSC were implanted into B6 mice [17]. This was associated with higher CD8^+^ T-cell numbers and increased levels of the Th1 cytokine IFNγ in the implant. Emphasizing the role of the T-cell response, implantation of allogeneic MSC in T-cell-deficient mice resulted in abundant bone formation [17]. This finding is in accordance with Liu et al., showing impaired osteogenic differentiation of MSC treated with IFNγ because of IFNγ-induced Runx2 downregulation [21]. Furthermore, they also reported a negative effect of infused pan T cells, purified CD4^+^, and CD8^+^ T cells on bone formation by BM-derived MSC. The association of increased CD8+ T cells with impaired bone formation has been confirmed by others. Reinke et al. reported impaired healing of transverse osteotomies in mice after adoptive transfer of CD8^+^ T cells, whereas depletion of CD8^+^ cells accelerated the healing process [22]. This is in accordance with the observation that fracture healing is accelerated in immunodeficient RAG1^−/−^ mice; however, the quality of the newly formed bone was inferior in such mice [23]. In approaches using xenogenic cells, application of hMSC led to inferior bone formation compared to autologous rabbit [16] or sheep MSC [15]. In both cases, no adverse immune reaction towards the xenogenic cells with respect to leukocytosis or C-reactive protein levels was reported [15,16].

There are also reports of successful bone regeneration using allogeneic MSC. Berner et al. reported similar bone regeneration by autologous and allogeneic MSC in an ovine model [14]. Additionally, the white blood cell count in venous blood as a measure of inflammation was not different between the treatment groups. However, it should be noted that the MSC-induced bone formation was considerably inferior to the gold standard autologous bone graft. A recent study reported equally efficient regeneration of mandibular defects in beagles using tissue-engineered bone with allogeneic or autologous canine MSC [24]. The authors noted a transient immune response with increased T-cell numbers and elevated cytokine levels in allogeneically treated dogs; however, this reaction was insufficient to suppress bone formation. Interestingly, the authors also observed significantly attenuated bone formation at four and eight weeks after implantation of the tissue-engineered allogeneic bone in an ectopic implantation site, although this effect was no longer found 12 weeks after implantation [24].

The question of whether the implanted MSCs themselves directly contribute to bone formation by producing the bone matrix or whether the cells have an indirect effect is frequently not considered. In the present study, we found that the implanted hMSC did not produce the collagenous bone matrix, because staining for human-specific type-I collagen was negative, whereas there was an intense signal when staining for murine Col1α2. This result is a strong indicator of the indirect action of the implanted autologous MSC. This presumption is strengthened by the observation that cells embedded in the bone or cartilage matrix were negative for both hb2m and Col1α2, and that cells embedded in the cartilage matrix produced murine Col1a2. Others suggest a more direct contribution. Arinzeh et al. described the presence of implanted fluorescently labelled (allogeneic) MSCs within newly formed bone tissue four weeks after implantation in a segmental defect in dogs, suggesting the osteogenic differentiation of the implanted cells [13]. After eight weeks, the fluorescent signal could only be found in the connective tissue within the implant, leading to the speculation that the osteogenically differentiated cells had undergone apoptosis and were replaced by host cells, or that the dye had diluted out. In cranial defects, implanted allogeneic adipose-derived stem cells were detected embedded in the regenerated skull bone as osteocytes, indicating intramembranous ossification by the cells [12]. Ultimately, the mode of contribution of the implanted cells remains unclear.

To unravel why bone formation might be diminished in mice treated with allogeneic hMSCs compared to autologous hMSCs, we analyzed cell proliferation by staining for Ki67 and angiogenesis by staining for VEGF, Cyr61, and CD31. We did not detect differences in Ki67 staining between autologous and allogeneically treated mice; however, cells embedded in the collagen gel matrix appeared largely negative, thus indicating no recent proliferative activity. Because vascularization is essential for ossification, we also analyzed angiogenic factors VEGF and Cyr61, and the formation of vascular structures. VEGF and Cyr61 were both found to be necessary for successful bone healing, because a blockade of VEGF [25] or Cyr61 [26] by inhibitory antibodies or genetic ablation [27] led to an impaired healing outcome. We found no differences in marker expression on day 10, indicating no cell-intrinsic deficits. On day 35, there was a significant reduction in CD31^+^ cells in allogeneic-treated animals, that is, significantly fewer vessels. The underlying cause of this difference remains unclear. As stated above, until day 10, there were no differences in angiogenic markers, so that the processes early in the post-operative phase do not appear to differ between autologous and allogeneic treatments. Which processes cause the impaired bone formation later on remains to be clarified.

In conclusion, our data show an inferior efficacy of allogeneic MSC to regenerate large bone defects compared to autologous MSC in a humanized mouse model; thus, allogeneic MSC currently do not appear to be an equivalent alternative to autologous treatment.

## 4. Materials and Methods

### 4.1. Animal Model and Husbandry

The experimental procedures were performed according to the national and international regulations for the care and use of laboratory animals and were approved by the Local Ethics Committee (Germany, Regierungspräsidium Tübingen, Reg. 1000 (approved 16 March 2010) and Reg. 1230 (approved 31 October 2015)). The mice were housed in isolated ventilation cages in groups of up to five mice with a 14-h light, 10-h dark cycle at 23 °C and 55 ± 10% humidity. Water and chow were available *ad libitum*.

### 4.2. Generation of Humanized Mice

Six- to eight-week-old male NOD/scid IL2Rγ_c_^null^ (NSG) mice were sub-lethally irradiated (200–260 cGy) and transplanted with 1 × 10^6^ purified human CD34^+^ hematopoietic stem cells within 24 h after irradiation. To ensure ideal conditions for the transplanted stem cells to engraft, the mice received intraperitoneal injections of 20 µg recombinant human IL-7 once weekly until stable engraftment was achieved. During the first four weeks after transplantation, antibiotics (10 mg/kg Enrofoxacin, Baytril^®^, Bayer, Leverkusen, Germany) were delivered via the drinking water. Mice with at least 3% human CD45^+^ cells in the peripheral blood 20 weeks after transplantation of hCD34^+^ were included in the bone-regeneration study.

### 4.3. Cell Culture and Preparation for Local Delivery

MSC for local implantation were isolated from BM aspirates of either the same donor as the hematopoietic stem cells (autologous set-up) or from another donor (allogeneic set-up, Figure 1). The cells were cultivated in Dulbecco’s Modified Eagle Medium (Lonza, Basel, Switzerland) supplemented with 8% platelet lysate (German Red Cross Blood Transfusion Service, Ulm, Germany), 80 IU heparin sulfate (Ratiopharm, Ulm, Germany), 1 mM l-glutamine, 100 U/mL penicillin, and 100 µg/mL streptomycin at 37 °C under 8.5% CO_2_ and saturated humidity. Cells up to passage 5 were used for transplantation.

One day prior to surgery, the MSC were harvested using trypsin/ethylendiamintetraacetic acid (EDTA) and seeded in a two-component collagen type-I gel (6 mg/mL collagen type-I, Amedrix GmbH, Esslingen, Germany) at a density of 200,000 MSC per 50 µL gel. For this, the MSC were resuspended in neutralization buffer and carefully mixed with collagen type-I-solution. The gels were allowed to polymerize for 15 min at 37 °C before serum-free medium was added. Before implantation into the bone defect, the collagen constructs were washed three times in phosphate-buffered saline to remove residual medium.

### 4.4. Generation of Critically Sized Bone Defects

To assess the intrinsic healing capacity of the humanized mice, we created 0.5-mm osteotomies and analyzed the healing outcome after 35 days. To compare the efficacy of autologous and allogeneic MSC to regenerate bone, 1-mm critically sized bone defects were created in the femur of the humanized NSG mice 20 weeks after transplantation. Briefly, the mice were anesthetized using 2–2.5 vol % isoflurane/oxygen. After a lateral approach to the femur, an external fixator (stiffness 18 N/mm^2^, MouseExFix, RISystem, Davos, Switzerland) was fixed to the cranio-lateral side of the femur using four mini-Schanz screws [28]. A 1-mm bone segment was excised from the mid-diaphysis using a Gigli wire saw (RIsystem). The defect was either left empty or filled with collagen gel (Amedrix) seeded with either autologous or allogeneic MSC. A cohort of mice was treated with cell-free collagen gel. The wound was closed in two layers. Prior to surgery, the mice received subcutaneous injections of antibiotics (clindamycin-2-dihydrogenphosphate, 45 mg/kg, Clindamycin, Ratiopharm) and analgesics (tramadol-hydrochloride, 15 mg/kg body weight, Tramal^®^, Gruenenthal, Aachen, Germany). For post-operative pain treatment, the analgesic was also applied via the drinking water (25 mg/L) until the third day after surgery.

### 4.5. Sample Collection and Processing

The mice were euthanized three, 10, or 35 days after surgery by blood withdrawal from the *Vena cava inferior* under deep general anesthesia. The right femurs were harvested, fixed in 4% neutral-buffered formaldehyde solution for 48 h, decalcified in 20% EDTA (pH 7.2–7.4) for 10–12 days, and embedded in paraffin. Some of the femurs (two for autologous, three for allogeneic hMSC) harvested after 35 days were processed for undecalcified histology and embedded in methyl methacrylate.

Sections of 6 µm thickness from decalcified femurs were stained using safranin-O/fast green, while non-decalcified sections were stained using von Kossa/van Gieson staining.

### 4.6. µCT Analyses

Before histological processing, femurs harvested after 35 days were scanned in a micro-computed tomography device (SkyScan 1172, Bruker, Kontich, Belgium) with a resolution of 8 µm/pixel at a peak voltage of 100 kV and 100 mA. The former bone defect was manually segmented using CTAnalyser (Bruker). For mineralized bone, a standardized threshold of 25% of the attenuation of the cortical bone was applied. Standard parameters defined by the American Society of Bone and Mineral Research were analyzed.

### 4.7. Multiplex Cytokine Analyses

Blood was collected in microvettes containing clotting activator (Sarstedt, Nümbrecht, Germany) and centrifuged at room temperature (RT) for 10 min at 2800 rpm. Serum was stored at −80 °C until used. Using a mouse multiplex cytokine assay (ProcartaPlex™, eBioscience, Frankfurt, Germany), the levels of TNF-α, IL-1β, IL-6, IFN-γ, MCP-1, MIP-1a, CXCL-1, IL-4, IL-10, and IL-13 were determined on a Bio-Plex 200 (Bio-Rad, Hercules, CA, USA). Cytokine levels were calculated automatically using standard curves (Bio-Plex Manager™ software, Bio-Rad).

### 4.8. Immunohistochemical Staining

For immunohistochemical analyses, sections were deparaffinised and rehydrated. Antigen-retrieval was performed by immersion in 0.1 M citric acid buffer pH 6 at 95 °C for 20 min. Nonspecific binding sites were blocked using 10% normal serum in tris-buffered saline pH 7.2 with 0.1% triton X-100 (Sigma-Aldrich, Taufkirchen, Germany) for 1 h at RT. Sections were incubated overnight a 4 °C with the respective antibody or isotype-control (ChromPure goat-IgG or rabbit-IgG, Jackson Immunoresearch, Ely, UK). Incubation with a biotinylated secondary antibody was performed at RT for 45 min to 1 h. The following antibodies were used: anti-human β2-microglobulin (hb2m) (1:700, DAKO, Hamburg, Germany), anti-human CD8a (1:100, OriGene, Rockville, MD, USA), anti-murine CD8 antibody (1:500, Bioss, Woburn, MA, USA), anti-Runx2 (1:50, Cell Signaling, Danvers, MA, USA), anti-murine CD31 (PECAM, 1:10, DAKO), anti-VEGF (1:800, Abcam plc, Cambridge, UK), anti Cyr61 (1:500, R&D Systems, Minneapolis, MN, USA), anti-murine Col1α2 (1:500, antibodies online), anti-human Col1α2 (1:200, antibodies online), and anti-murine F4/80 antibody (1:500, Bio-Rad AbD Serotec GmbH, Puchheim, Germany). For hb2m horseradish peroxidase-linked streptavidin and subsequent ACE single solution (both Zytomed, Berlin, Germany) were applied for visualization. Staining for Runx2 and human and murine Col2α1 were visualized using Vectastain^®^ Elite^®^ ABC kit and NovaRed™ substrate (both Vectorlabs, Burlingame, CA, USA). The sections were counterstained with hematoxylin and mounted.

### 4.9. Histomorphometric Analysis

Evaluation of the defect region was performed using a light microscope (Leica DMI6000 B, Leica Microsystems, Wetzlar, Germany) and image analysis software (Leica MetaMorph^®^ software). The defect region was analyzed for bone, cartilage and fibrous tissue contents. The sections were digitalized using 50× magnification. The digitalized images were then used for the analysis. Using the MetaMorph^®^ Software and a customized plug-in, the area of the former defect region was outlined as well as the outlines of bone and cartilage areas. The area was automatically calculated by the program and the soft tissue area was determined by subtracting bone and cartilage from the defect area. The relative proportions in relation to the defect area were calculated using Microsoft Excel.

### 4.10. Statistics

Statistical analyses were performed using GraphPad Prism 6 (GraphPad Software Inc, La Jolla, CA, USA). Data were tested for normality using the D’Agostino–Pearson omnibus normality test or a Q–Q plot. For comparison of two normally distributed groups, Student’s *t*-test was applied; three groups were compared using one-way analysis of variance with Tukey’s correction for multiple tests. *p* ≤ 0.05 was considered statistically significant.

## Figures and Tables

**Table 1:** Cytokine/chemokine levels in pg/mL of serum obtained 3 days after surgery. Data are presented as the mean ± SD.

pg/mL	Empty (*n* = 3)	Autologous (*n* = 2)	Allogeneic (*n* = 8)
CXCL1	19.2 ± 2.3	19.1 ± 4.5	20.0 ± 2.2
MCP-1	66.7 ± 14.0	88.6 ± 38.5	84.3 ± 25.8
IL-6	16.1 ± 12.4	23.4 ± 16.4	39.7 ± 20.0
TNF-α	1.5 ± 0.2	1.2 ± 0.4	1.6 ± 0.1
IL-4	0.2 ± 0.03	0.2 ± 0.05	0.2 ± 0.06
